# Recombinant BCG: Innovations on an Old Vaccine. Scope of BCG Strains and Strategies to Improve Long-Lasting Memory

**DOI:** 10.3389/fimmu.2014.00152

**Published:** 2014-04-07

**Authors:** Adeliane Castro da Costa, Sarah Veloso Nogueira, André Kipnis, Ana Paula Junqueira-Kipnis

**Affiliations:** ^1^Department of Microbiology, Immunology, Parasitology and Pathology, Instituto de Patologia Tropical e Saúde Pública, Universidade Federal de Goiás, Goiânia, Brazil

**Keywords:** rBCG, tuberculosis, vaccine, protection, long-term memory, strain differences

## Abstract

Bacille Calmette–Guérin (BCG), an attenuated vaccine derived from *Mycobacterium bovis*, is the current vaccine of choice against tuberculosis (TB). Despite its protection against active TB in children, BCG has failed to protect adults against TB infection and active disease development, especially in developing countries where the disease is endemic. Currently, there is a significant effort toward the development of a new TB vaccine. This review article aims to address publications on recombinant BCG (rBCG) published in the last 5 years, to highlight the strategies used to develop rBCG, with a focus on the criteria used to improve immunological memory and protection compared with BCG. The literature review was done in April 2013, using the key words TB, rBCG vaccine, and memory. This review discusses the BCG strains and strategies currently used for the modification of BCG, including: overexpression of *Mycobacterium tuberculosis* (Mtb) immunodominant antigens already present in BCG; gene insertion of immunodominant antigens from Mtb absent in the BCG vaccine; combination of introduction and overexpression of genes that are lost during the attenuation process of BCG; BCG modifications for the induction of CD8+ T-cell immune responses and cytokines expressing rBCG. Among the vaccines discussed, VPM1002, also called rBCGΔureC:hly, is currently in human clinical trials. Much progress has been made in the effort to improve BCG, with some promising candidates, but considerable work is still required to address functional long-lasting memory.

## Introduction

Tuberculosis (TB) is an infectious disease caused by *Mycobacterium tuberculosis* (Mtb), an intracellular pathogen that, after infecting a host, can cause disease or latency. TB continues to kill some 1.3 million people annually and 2 billion people worldwide are infected with Mtb (1, 2). The attenuated *Mycobacterium bovis* strain, known as Bacille Calmette–Guérin (BCG), is currently the only TB vaccine approved for human use, but its protective efficacy remains doubtful (3, 4). BCG was initially obtained from a virulent strain and was developed in France between 1908 and 1921 by Albert Calmette (1863–1933) and Camille Guérin (1872–1961). Although BCG is efficient in some regions of the world, such as in Alaskan American Indians region (5, 6), the protection conferred by BCG varies between 0 and 80% (7–9), although it has efficacy in protecting children from severe forms of TB.

To achieve BCG attenuation, more than 10 years of research with more than 230 serial passages were performed *in vitro* (10). This attenuation promoted genomic deletions, that together with the evolution of *M. bovis*, resulted in 16 genomic regions of differentiation (RD1–RD16, plus nRD18), when compared with the Mtb genome (11, 12). Regarding the region of differentiation lost during attenuation, RD1 is a DNA segment comprising 9.5 kb, which was deleted in all other BCG strains, that encodes T-lymphocyte epitopes such as ESAT-6, CFP-10, Rv3873, and PPE protein among others (13); RD2 is a 10.7 kb DNA segment that encodes many proteins including Mpt64 and CFP-21 (12); RD14 is a 9.1 kb section of DNA encoding proteins of the PE-PGRS and Rv1771 families (gulonolactone dehydrogenase) (14); RD16 is a 7.6 kb DNA section encoding Rv3405 that is responsible for colony morphology characteristics and the formation of cell membrane constituents (15); and nRD18, a 1.5 kb segment containing genes encoding SigI, an alternative RNA polymerase sigma factor, that was only lost in the strains BCG Pasteur, Phipps, Frappier, Connaught, and Tice (12). During the BCG attenuation process and the years that followed, more than 14 sub-strains emerged: BCG Russia (ATCC 35740), BCG Moreau/Rio de Janeiro, BCG Tokyo, BCG Sweden, BCG Birkhaug (ATCC 35731), BCG Denmark 1331 (ATCC 35733), BCG China, BCG Prague, BCG Glaxo (ATCC 35741), BCG Tice (ATCC 35743), BCG Frappier (ATCC 35735), BCG Connaught, BCG Phipps (ATCC 35744), and BCG Pasteur 1173 (16). They are distributed worldwide and have been used for vaccine development to prevent TB. The main concern is that BCG administration does not provide a reliable protection for adults in the developing world, protecting just against the main causes of infant TB, TB meningitis, and miliary TB (2).

To address the evolution of new recombinant BCG (rBCG) vaccines, the immunological status goal for such a vaccine should be defined. This is a controversial issue as there is no consensus as to what is the ideal immune memory phenotype that can confer protection. For instance, in animal models such as mice, both Mtb infection or BCG vaccine induce increased levels of lung CD4+ effector T cells (phenotype CD44^hi^CD62L^lo^CCR7^lo^) as well as memory cells. The current memory cell phenotypes accepted are effector memory T cells (TEM, CD44^hi^CD62L^lo^CCR7^lo^) and central memory T cells (TCM, CD44^hi^CD62L^hi^CCR7^hi^) (17–19). An important cornerstone for protection against TB is interferon (IFN)-γ production by T cells (20, 21), a cytokine crucial for stimulation of the microbicidal functions of macrophages. More recently, some authors have proposed that the desired protective memory against TB infection should have a central memory characteristic, with polyfunctional ability to produce IFN-γ, tumor necrosis factor (TNF)-α, and interleukin (IL)-2 cytokines (22) or a balance between IFN-γ and IL-17 levels to avoid excessive pathology (23).

The ultimate goal of a vaccine is its protective use in humans; consequently, the characterization of memory T cells in humans is also crucial. The major cell surface biomarker expression pattern for human memory T cells with an effector phenotype is CD45RA^hi^, CD45RO^neg^, and CCR7^neg^, while for central memory T-cell populations it is CD45RA^hi/low^, CD45RO^neg^, and CCR7^pos^. A follow-up study conducted among children vaccinated with BCG showed that specific memory T cells were stimulated and present in the peripheral blood of those individuals for at least 52 weeks following vaccination (24). It is interesting to observe that the induced memory cells were polyfunctional (IFN-γ, TNF-α, and IL-2). Although several studies have characterized the memory phenotypes induced by BCG, the direct association of memory T-cell populations with TB protection is still not well-established. Recently, a long-lasting T-cell memory population expressing CD127 was associated with Mtb infection and may correlate with protection shown by some exposed individuals (25). For this review, we consider the memory T-cell population as those that have the following phenotypes: CD4^+^ CD44^hi/low^ CD62L^lo^ or to be antigen-specific CD4+ IFN-γ producing cells.

A significant limitation of TB vaccine development and testing is the lack of an optimal animal model that truly reflects human TB disease and the progress of immune responses. While there are several new vaccines being developed in different laboratories, there is a diversity of animal models (mice, rabbits, guinea pigs, non-human primates) and disease outcomes being used by different laboratories, preventing an adequate comparison between them. In addition, there is no consensus on the protocol to be used for vaccination and challenge, with different routes of immunization/infection, doses, BCG and Mtb strains, and time periods being used. Short period of time between vaccination and challenge does not allow full immunological memory development, thus generating a bias in the correlation between memory T-cell phenotype and protection. The most accepted method for evaluating protection is the determination of the bacterial load following challenge of vaccinated animals compared to non-vaccinated infected controls. Although colony-forming unit (CFU) counting is a widely used method, the organs used to assess the bacterial load vary among researchers, and make it difficult to establish comparisons. Given these many different parameters, in this review protection conferred by the different rBCG vaccines was considered when an overall significant reduction of the bacterial load compared with wild type BCG was achieved.

The factors that determine the induction of immunological memory related to BCG are not well-understood. Some assumptions are directed to the characteristics of the BCG sub-strains, which exhibit genotypic and phenotypic differences after the attenuation process as well as distinct residual virulence levels, the number of epitopes of each BCG strain, or the recombination strategy used for the development of a new vaccine (26, 27). According to the research tools employed in this study, of all sub-strains originated after this process the strains most frequently tested over 5 years were BCG Tokyo (BCG Japan), BCG Tice, BCG Danish (BCG Denmark/BCG SSI 1331), BCG Pasteur, BCG China (BCG Shanghai), and BCG Prague. It is also hypothesized that generation of the immune response and eventually the outcome of vaccination could be influenced by the type of genetic strain background used. However, pre-clinical animal and human data have demonstrated that different strains of BCG confer the same level of protection (28, 29).

The main strategies used to develop new vaccines are based on the formulation of subunit vaccines; the production of non-recombinant viral vector vaccines that can be used as a BCG prime boost, and the construction of rBCG, which could confer similar protection with a better induction of memory than BCG. Methods to construct rBCG include overexpression of promising Mtb immunodominant antigens expressed by BCG, such as α-crystallin: HspX protein and antigen 85 (Ag85) complex proteins (Ag85A, Ag85B, and Ag85C) (30); insertion of Mtb immunodominant antigens absent on BCG, such as those encoded by RD1, RD2, RD3, RD14, RD15, RD16, and nRD18 genes (27); combination of overexpression with reintroduction of genes lost during BCG attenuation; and BCG modification to induce CD8+ T immune response proteins and cytokines (Tables 1 and 2).

**Table 1:** **BCG sub-strains genetic background used for recombinant BCG vaccines development and ability to induce memory and protection against tuberculosis**.

Sub-strains	RDs^a^	Epitopes	No^b^	Protection better than BCG?^c^	Memory?^d^	Reference
Tokyo/Japan	RD1	359	3	Yes	Yes	(31 , 32 )
				Yes	No	(33 )
Pasteur	RD1, RD2, RD14, nRD18,	331	6	Yes	No	(34 )
				No	Yes	(35 )
				No	No	(36 –38 )
Danish/Denmark	RD1, RD2	329	11	Yes	Yes	(39 )
				No	Yes	(40 )
				Yes	No	(41 –45 )
				No	No	(46 –48 )
Tice	RD1, RD2, nRD18	328	2	Yes	Yes	(49 )
				Yes	No	(50 )
China/Shanghai	RD1, RD2	321	6	Yes	No	(51 )
				No	No	(52 –56 )
Prague	RD1, RD2	318	3	Yes	No	(23 )
				No	No	(57 , 58 )

*^a^RD, region of difference*.

*^b^Number of publications in the last 5 years*.

*^c^Protection was evaluated by CFU analyses and considered when the bacterial load of challenged animals were lower than wild type BCG-vaccinated animals*.

*^d^Memory was defined as CD4^+^ CD44^hi^ CD62L^lo^ or CD4+ IFN-γ producing T cells specific immune responses*.

**Table 2:** **Description of strains and antigens used in the papers visited for this review**.

Reference	Model	Strain	Antigen	Challenge	Protection^a^
(31)	Mice	BCG Tokyo	rBCG1:Ag85B–CFP10(rBCG1)/BCG2:Ag85B–CFP10–IL-12 (rBCG2)	No	> (*Ex vivo*)
(49)	Human	BCG Tice	rBCG30 (Ag 85B)	No	NE
(46)	Guinea pigs	BCG Danish	rBCG–E6 (ESAT-6)	50–100 bacilli of *Mtb*	>
(41, 42)	Guinea pigs	BCG Danish	rBCG*acr*	50–100 bacilli of *Mtb*	>
(39)	Mice	BCG Danish	BCG:HspX/rBCG:85B	10^6^ CFU of *Mtb*	>
(53)	Mice	BCG China	rBCG–AE	10^6^ CFU of *Mtb*	<
(58)	Mice	BCG Prague	rBCGureC:hly or rBCGureC:hly	10^2^ CFU of *Mtb*	=
(45)	Mice	BCG Danish	BCG: rBCG–Ag85B–Mpt64–Mtb8.4	10^6^ CFU of *Mtb*	>
(47)	Mice	BCG Danish	rBCG:Ag85B–ESAT-6–Rv2608	No	NE
(33)	Monkey	BCG Tokyo	rBCG–Ag85A	3000 CFU of *Mtb*	>
(48)	Monkey	BCG Danish	rBCG AFRO-1	No	NE
(55)	*In vitro*	BCG China	rBCG:Quimera 85B + ESAT-6	No	NE
(34)	Mice	BCG Pasteur	rBCG:PE–MPT64/rBCG/HSP60MPT64	~200 CFU of *Mtb*	>
(23)	Mice	BCG Prague	rBCGΔureC:hly+	200–400 CFU of *Mtb*	>
(51)	Mice	BCG China	rBCG: Ag85A/rBCG:Ag85B/rBCG:Ag85A–Ag85B	10^6^ CFU of *Mtb*	>
(52)	Mice	BCG China	rBCG: Ag85A–ESAT-6/rBCG: Ag85A/rBCG: ESAT-6	No	NE
(40)	Monkey	BCGDanish	AFRO-1, Ag85A, Ag85B e TB10.4.	500 CFU of *Mtb*	>
(37)	Mice	BCG Pasteur	rBCGs:BCG:Ag85c, BCG:INV, BCG:PPE, BCG:FBP e BCG:CFP	20 bacilli of *Mtb*	=
(57)	Mice	BCG Prague	rBCGΔureC:hly+)	No	NE
(38)	Mice	BCG Pasteur	rBCG: pHspX–Ag 85B	100 bacilli of *Mtb/lung*	=
(56)	Mice	BCG China	rBCGs:BCG:GM-CSF/BCG:ESAT-6/BCG:GMCSF–ESAT-6	No	NE
(32)	Mice	BCG Tokyo	rBCG–85B–IL-15/rBCG–85B	2 × 10^5^ CFU of *Mtb*	=
(44)	Guinea pigs	BCG Danish	rBCG–85C	500 bacilli of *Mtb*	>
(59)	Human	BCG Danish	BCG **Δ**ureC:hly Hm^R^	No	NE

*^a^Protection was evaluated by CFU analyses and considered when the bacterial load of challenged animals were lower than wild type BCG-vaccinated animals; >, superior than BCG; =, protection similar to BCG; –, less protection than BCG; NE, not evaluated*.

*References published and indexed in PubMed from 2008 to April 2013*.

Therefore, the aim of this review was to analyze which factors associated with rBCG could induce long-lasting memory and promote better protection compared with conventional BCG.

## Does BCG Epitope Number Influence the Induction of Memory and Protection of rBCG Vaccines?

As stated by Zhang et al. (27), the number of epitopes in a particular strain can be important for the development of an enhanced vaccine that could replace BCG. According to this hypothesis, a strain such as BCG Tokyo has the potential to induce a better immune response compared with conventional BCG because it comprises 359 epitopes that can be recognized by lymphocytes (27). To verify if there is sufficient data to support this hypothesis in the last 5 years, we selected criteria as summarized in Table 1. The different rBCG vaccines were compared according to their ability to improve protection by reducing the bacterial load relative to wild type BCG and to generate specific memory CD4+ T cells. Three different published studies using rBCG Tokyo, which contains a high number of epitopes, showed better protection than BCG, which was associated with the recombinant generation strategy: overexpression and reintroduction of lost genes, as well as the presence of cytokines (Table 1). Nevertheless, the strain that has been most widely used in the last 5 years is BCG Danish (BCG Denmark/BCG SSI 1331), which has approximately 329 epitopes, and provides improved protection and long-lasting memory when associated with the overexpression of Mtb antigens. Two rBCG Tice (328 epitopes) vaccine constructions also demonstrated good induction of protection but only one induced memory. Based on those publications, it appears that the genetic background of the BCG strains (number of epitopes) does not have a major role in inducing/improving protection and memory.

Contrary to Zhang et al. (27), other studies support the idea that recombinant antigen selection expressed by BCG, and not the BCG strain background, is the significant point to be considered in the construction of an improved vaccine with enhanced induction of memory and protection (32, 34, 46). Moreover, it appears that overexpression of certain antigens in rBCG are critical for enhanced induction of memory and protection compared with BCG.

One limitation of this study is that when analyzing studies only published in the last 5 years, the conclusions might be biased because important work addressing whether the BCG strain background (epitope number) or selected antigen were important for enhanced memory and protection may have been published earlier.

## Does the Quantity of Mtb Antigens Incorporated in BCG Result in Greater Protection and Memory Development?

### rBCG vaccines super expressing Mtb immunodominant antigens

An important strategy used for the construction of a new TB vaccine is the development of an rBCG super expressing Mtb immunodominant antigens, such as proteins from the Ag85 Complex, HspX protein, and the association of both proteins in one vaccine construction, which represents a favored approach for TB vaccine construction (44).

Some of the most important antigens used to construct rBCG vaccines are those from the Ag85 Complex that consists of Ag85A (Rv3804c), Ag85B (Rv1886c), and Ag85C (Rv0129c), encoded by fbpA, fbpB, and fbpC2 genes, respectively, and with molecular weights between 30 and 32 kDa (60). Proteins of Ag85 complex have mycolyltransferase activity, thus they play a role in mycolate production and construction of the Mtb cell wall, which is important for maintaining Mtb integrity and pathogenesis (61).

The protein Ag85B, used for construction of the vaccine rBCG:30 (r30–Ag85B), generated protection in guinea pigs after challenge with Mtb (62, 63). A phase I clinical trial of rBCG:30 in human volunteers induced central and effector memory CD4 and CD8 T cells specific for Ag85B (49). Currently, this vaccine is no longer being tested on humans. The same antigen was used by Tullius et al. (50) who developed a mutant rBCG, rBCG(mbt)30, resulting in a strain unable to synthesize mycobactin and exoquelin molecules that are essential for iron acquisition. The vaccine induced greater protection than conventional BCG. Another approach was to design an rBCG pantothenate auxotroph, rBCG(panCD)30. Both vaccines, rBCG(mbt)30 and rBCG(panCD)30, were attenuated to a higher degree than BCG and induced potent protective and cell-mediated immunity in guinea pigs (50). These vaccines may have the potential to provide a safe alternative for HIV positive individuals since BCG is not indicated for use in immunocompromised individuals.

rBCG:30 and other vaccines overexpressing Ag85B were better at conferring protection and memory than BCG. Ag85B (30 kDa) is the most abundant protein of the Ag85 complex, and is the most abundant extracellular protein of Mtb, responsible for nearly one-quarter of the total extracellular protein in broth culture (64). In addition, Ag85B has a high affinity for T-cell recognition, can induce a type Th1 immune response with IFN-γ production, and has a good protective capacity when used in DNA vaccine strategies (65).

Ag85C is also a major secretory protein and immunodominant antigen, being strongly recognized by sera from TB patients. Indeed, it is responsible for almost 40% of the mycolate content of Mtb and its mycolyltransferase activity cannot be substituted by Ag85A or Ag85B (66). For this reason, Jain et al. (44) developed an rBCG expressing Ag85C under the transcriptional control of mycobacteria promoters. Reduced granulomatous infiltration and granuloma formation were observed when compared to a group immunized with BCG and the protection (reduced bacterial load in lungs and spleen compared with ancestor BCG) was associated with reduced levels of IFN-γ, TNF-α, IL-12, and TGF-β mRNA compared to BCG. However, high levels of inducible nitric oxide synthase (iNOS) were observed compared with BCG. Furthermore, previous studies with DNA vaccines using Ag85C demonstrated the reduced production of IL-2 and IFN-γ with insufficient protection when animals were challenged with *M. bovis* BCG (67). Hence, it is important to stress that both the type of antigen and its expression in a suitable vector (BCG itself) is important to confer good protection status. The study by Lozes et al. used the BCG Danish strain, which may have contributed to the disappointing results. Unfortunately, the study did not provide information regarding the ability to generate memory cells.

Ag85A is strongly recognized by T lymphocytes to induce IL-2 and IFN-γ production (67). Immunization of mice and guinea pigs with rBCG:Ag85A promoted the reduction of pulmonary pathology severity and increased protection in lungs and spleen against infection (68). Consequently this vaccine was also tested in *Macaca mulatta*, and, after challenge, the group immunized with rBCG:Ag85A developed light to moderate pneumonia, while the non-vaccinated group developed multilobar pneumonia, lymphadenopathy, and atelectasis. In addition, its protective capacity was previously appraised in a DNA vaccine system with Ag85A (69). It was shown that rBCG–Ag85A induced higher protective efficacy than the parental BCG Tokyo strain (33). In that study, a strategy of over expressing the antigen in addition to the use of a BCG strain containing more natural epitopes was employed (Table 1). Hence, this could justify the potential of this vaccine for further studies to measure memory induction.

The construction of rBCG expressing single proteins resulted in promising results. Following those studies, significant progress was made with the construction and testing of recombinant fusion proteins, combining two or more protein coding regions from one or more Mtb proteins, because the combined use of antigens might enhance protective efficacy compared with rBCG expressing only one antigen.

To analyze this hypothesis, Wang et al. developed three vaccine constructions: rBCG:Ag85A (A), rBCG:Ag85B (B), and rBCG:AB, which were used to immunize mice. The vaccine containing fusion antigens, rBCG:AB, showed better protection after challenge with Mtb, when compared with BCG or rBCG expressing Ag85A or Ag85B alone. Six and 24 weeks after vaccination, splenocytes of mice immunized with rBCG:AB stimulated with specific antigen secreted more IFN-γ than splenocytes from mice immunized with the other rBCG (51). Regrettably, no memory response was evaluated in that study, probably because memory induction was already known for rBCG:Ag85B (49). However, no studies using rBCG expressing Ag85A have assessed memory responses, so it would be meaningful to verify whether Ag85A could contribute to enhanced memory responses. Although an approach using recombinant fusion proteins is valuable for protection against challenge with Mtb, additional studies regarding the induction of functional, long-lasting memory is also required for the development of new vaccines.

Another antigen frequently used for recombinant expression in BCG is the HspX protein (Rv2031c, also known as α-cristalin, molecular weight 16 kDa), a heat shock protein encoded by the *acr* gene (70). This protein is abundantly produced during the latent or persistent Mtb metabolic condition. Shi et al. developed a rBCG over expressing the immunodominant Mtb antigen, HspX (rBCG:X) and demonstrated that rBCG:X provided enhanced and longer lasting protection against Mtb infection than BCG, as evidenced by high levels of IFN-γ production, low bacterial load in tissues, and reduced lung pathology. This was associated with elevated levels of anti-HspX antibodies during week 6 and 24 (168 days) after rBCG:X immunization, indicating that BCG:X might persist longer *in vivo* than BCG (39).

Additionally, results obtained by Shi et al. demonstrated that expression of HspX by BCG could improve its biological effects, which might explain the higher expression of Ag85B in the supernatant and lysate of cells after infection with rBCG:X compared with that by BCG (39). This theory was also corroborated by Kong et al. (38) who constructed an rBCG expressing Mtb Ag85B under the control of a HspX promoter. The expression and immune response to Ag85B was modulated by the HspX promoter. For example, rBCG:PhspX-85B induced intense specific Ag85B T-cell proliferation and IFN-γ production 3 weeks after infection. Increased cell proliferation and IFN-γ production was observed after 12 weeks indicating long-lasting cell-mediated immunity. Despite the intense induction of immune cell responses, the protection in lungs and spleen induced by this vaccine was similar to that by BCG. This indicated that in a model of Ag85B expression under control of a different promoter, there was no improvement in protective efficacy (38). Although Ag85C is responsible for more than 40% of the mycolate present in the mycobacteria cell wall (61), evidence suggests that Ag85B is critical for enhanced BCG induction of memory and protection (49, 50).

The use of fusion proteins has generated great expectations in the scientific community, nonetheless, the use of combined proteins yielded no better memory than BCG, according to the present accepted parameters, generating only better protection. The increased protection observed among the recombinant vaccines cannot be the only improvement desired for the development of a new vaccine, as vaccination of available animal models to study new vaccines to TB does not eliminate all Mtb from the tissues of challenged animals. Therefore, new definitive protection parameters are needed.

### Association of overexpression and reintroduction of antigens lost during the attenuation process

Some virulence regions, such as RD1, were lost during the BCG attenuation process. RD1 is absent in all BCG sub-strains, but present in virulent strains and clinical isolates of *M. bovis* and *M. tuberculosis*. The association of Mtb genes lost in the *M. bovis* attenuation process within rBCG has been used to improve vaccine efficacy (11). The collection of well-defined T-cell antigen epitopes has been a widely used strategy for the construction of new vaccines. This collection is based on the reintroduction of proteins whose gene regions were deleted during the attenuation process and include the 10 kDa culture filtrate protein (CFP-10, Rv3874), ESAT-6, PPE family protein (Rv3873), INV (Rv1474), and MPT64.

When evaluating the induction of immune responses, vaccine constructions containing antigenic epitopes have been the most successful, although most studies did not evaluate the protection or memory induced by these vaccines. Vaccine constructs using antigen epitopes were good inducers of antigen-specific Th1 (IFN-γ) immune responses, IgG2a production, and delayed type hypersensitivity responses compared with BCG. Conversely, some recombinant vaccines (BCG:CFP, BCG:FBP, BCG:PPE, and BCG:INV) showed protection similar to that of BCG (37). Only rBCG vaccines expressing MPT64 antigens fused to a PE antigen (HPE–ΔMPT64–BCG) showed superior protection than those immunized with BCG. This protection was associated with CD4 and CD8 T-cell induction and the emergence of a specific MPT64 T-cell clone (34). Despite use of the same BCG strain in the two studies, the differences in protection observed indicate the importance of antigen choice.

When using proteins of the Ag85 complex, Qie et al. (45) compared the protective efficacy of rBCG–AMM (BCG expressing Ag85B–MPT64190–198–Mtb8.4) with BCG. Animals vaccinated with rBCG–AMM generated more antigen-specific CD4 and CD8 T cells than those vaccinated with BCG and showed a more efficient response that protected mice challenged with H37Rv Mtb strain. Moreover, rBCG–AMM was superior to BCG in reducing the severity of disease in the target organs such as lungs and spleen, indicating that rBCG–AMM could be a potential vaccine candidate for further studies. Again, this vaccine was not evaluated for memory induction.

Some rBCG vaccines designed over the past 5 years combined the ability to generate strong immune responses to Ag85 proteins using the antigen ESAT-6 (47, 52, 55). Of these studies, only one addressed protection and memory development. Deng et al. (52) constructed an rBCG expressing the fusion protein Ag85A–ESAT-6 (rBCG–AE) and this vaccine induced more potent immunogenicity than native BCG in mice and induced a shift toward a Th1 type immune response with an increase in the ratio of CD4 and CD8 T-cell subsets. Thus, rBCG–AE elicited long-lasting and stronger Th1 type cell-mediated immune responses than BCG. They further evaluated the protective efficacy conferred by rBCG–AE against Mtb infection in BALB/c mice (53). An rBCG vaccine expressing ESAT-6 alone did not exceed the parental BCG vaccine for protection from Mtb H37Rv infection. The vaccine was developed using a BCG China strain, while others used BCG Tokyo, BCG Danish, or BCG Pasteur. As previously stated, vaccine construction over expressing proteins of the Ag85 Complex seem to have better protection efficacy than BCG, while rBCG–ESAT-6 induced protection similar or even inferior to BCG. However, using combined epitopes from proteins of the Ag85 complex or other proteins and ESAT-6 improved macrophage activation and antigen presentation (55), and strong humoral and cellular immune responses were induced (47), although protection or memory generation was not addressed.

The development of rBCG vaccines that re associated with or reintroduced genes lost during BCG attenuation appears to improve protection and memory most frequently when proteins from the Ag85 complex are associated with the fusion protein. This observation could be biased as Ag85 proteins are most frequently used in the development of rBCG vaccines. The combined results suggest that these genes are evolutionary maintained by Mtb to induce strong immune responses in animals and humans, independent of the type of BCG strain used. Most studies presented here did not evaluate functional memory, a crucial step for the development of a long-lasting protective vaccine.

### rBCG vaccine expressing mammalian cytokines and Mtb proteins

Cytokines play a central role in the immune system and have multiple effects on different immune cells. IL-2, for example, has been used for the treatment of some diseases, including TB, but toxicity related to high doses has restricted its use. A solution to that problem was the expression of rIL-2 and other cytokines by BCG (71). Another example is IL-15, an important cytokine that maintains survival and proliferation of CD8+ T cells with a memory phenotype (72). To develop new vaccines capable of improving BCG vaccination, some research strategies have included the use of rBCG expressing IL-2, IL-12, IL-15, and GM-CSF, among others.

Recombinant vaccines expressing cytokines induced effector polyfunctional CD8 T cells and CD4 T cells (producing IFN-γ, IL-2, and TNF-α), as well as humoral immune responses with increased specific IgG2a/IgG1 levels [(32) rBCG–Ag85B–IL-15; (31) BCG: Ag85B–CFP10–IL-12; (56) rBCG: GMCSF–ESAT-6]. Among the types of vaccines, those that expressed IL-15 and IL-12, were most successful as they induced CD8+ T (CD8+ CD44^hi^ CD62L^lo^) and CD4+ T (CD4+ CD44^hi^ CD62L^lo^) memory cells (31, 32).

Although these vaccines showed better protection than BCG, the rBCG–Ag85B–IL-15 vaccine was more promising because it generated a greater induction of memory CD8 T cells than memory CD4 T cells, in support of the theory that CD8 T cells rather than CD4 T cells are important for long-lasting protection against TB (32). It is important to note that despite the positive influence of IL-15 in inducing memory cells, *in vivo* administration after priming with rBCG followed by challenge with Mtb, did not induce increased numbers of CD8 T memory cells, a phenomenon only seen when IL-15 is expressed by rBCG (32).

Thus, the induction of CD8+ T cells and polyfunctional CD8+ and CD4+ T cells (producing IFN-γ, IL-2, and TNF-α) are responsible for the improvement of protection generated by rBCG while the secretion of ILs might play an important role in the proliferation and maintenance of memory T cells.

## BCG Modification: Induction of CD8 T Immune Responses

Mtb and BCG preferentially localize inside antigen presenting cell (APC) phagosomes, such as macrophages and dendritic cells. This localization dictates antigen traffic via MHC-II, which results in the preferential stimulation of CD4 T cells. CD8 T cytolytic lymphocytes (CTLs) are essential for the clearance of intracellular Mtb infection since CTLs kill cells and bacteria through secretion of cytolytic and antimicrobial effector molecules (perforin and granulysin). Mtb induced apoptosis in infected cells, resulting in vesicles that transport mycobacteria antigens, which can be captured by local dendritic cells that cross present MHC-I and MHC-II, that stimulate CD8 and CD4 T cells, respectively (73). It is also acknowledged that BCG is a weak inducer of apoptosis and thus activates CD8 T cells to a lesser extent (57, 73). Thus, in an attempt to improve BCG, rBCG vaccines have been developed to express listeriolysin (Hly) from *Listeria monocytogenes* (74) in the membrane, in combination with deletion of the Urease C (ureC) gene (rBCGΔureC:hly). One mechanism of BCG employs to survive phagosomes is pH neutralization through ureC activity. To induce apoptosis, Hly requires an acidic pH. The ureC mutant rBCG allows phagolysosome pH acidification to occur naturally (74). Using this vaccine protocol, Reece et al. (58) selected antigens based on their expression in response to nutrient deprivation (Rv2659c), hypoxia (Rv1733c), or disease reactivation (Rv3407) and transformed rBCGΔureC:hly with plasmids containing these antigens, rBCGΔureC:hly (pMPIIB01). The improved performance of this vaccine was demonstrated by a lower bacterial load in the spleen of infected mice (58). In addition, it induced Th-17, CD4+, and CD8+ T-cell responses and increased protection compared with BCG (23, 57). This vaccine is the most promising rBCG vaccine generated and it finished a phase I clinical trial for safety with great success. It is currently being tested in newborns in a phase II clinical trial (59). Although this vaccine aimed to improve T CD8 responses, the induction of specific CD4 T cells secreting IFN-γ as well as polyfunctional T CD4 responses were observed in vaccinated healthy humans.

In a similar approach to obtain a vaccine inducing increased CD8 T-cell responses, an rBCG, rBCG AFRO-1 (BCG expressing Ag85A, Ag85B, and TB10.4) was developed followed by two boosts with AERAS-402 [adenovirus vaccine 35 (rAd35) expressing Ag85A, Ag85B, and TB10.4]. AFRO-1 BCG expresses perfringolysin O, which allows BCG to escape to the cell cytosol, promoting antigen processing and presentation via MHC-I. After priming with rBCG AFRO-1, there was delayed but strong IFN-γ production 1 week after boost with AERAS-402, as well as strong proliferation of CD4 and CD8 T cells (48). This vaccine promoted longer survival and IFN-γ production; however, no difference in lung and spleen bacterial load between the groups vaccinated with BCG or AFRO-1 (also known as AERAS-422) was observed (43). Although a promising vaccine, AERAS-422 was terminated because of the development of shingles in some study participants that occurred during a phase I clinical trial (75).

The strategy of BCG modification for the induction of CD8 T specific immune responses has had a great impact, as the recombinant vaccine rBCGΔureC:hly is in a clinical trial^1^.

## Conclusion and Future Perspectives

Bacille Calmette–Guérin has been used for almost 100 years, with more than eight million doses used. However, TB incidence has shown a slow decrease during the last decade, mainly due to the increase of multi drug resistant strains and HIV co-infection (6). Two main cautions of BCG vaccine use are associated with its variable efficacy and immunity against Mtb infection resulting in a large pool of latently/persistently infected individuals. Furthermore, BCG might induce better protection among individuals from regions with lower environmental mycobacterial contaminations and lower TB rates. Development of a new vaccine or improvement of BCG to protect against TB is not an easy task, once the natural infection *per se* does not induce protection or long-lasting T or B functional memory cells since it does not avoid re-infection. It appears that the coevolution between mycobacteria and humans favors the mycobacteria. Over the past 5 years, several attempts were conducted to develop rBCGs (Table 2). Improvement of BCG remains the best choice for the rational design of a TB vaccine. This review sought to discuss recent TB studies advancing the rBCG strategy. The main purpose for developing rBCG is to design a vaccine capable of inducing long-lasting functional memory with protection similar or superior to that of BCG. In addition, BCG is a strong inducer of CD4+ T cells but it is an insufficient stimulator of CD8 T cells. The most effective rBCG vaccination strategies in animal models and human clinical trials to date were those that stimulated both CD4+ T and CD8+ T cells to produce Th1-associated cytokines and induce cytotoxic functions [(24, 76), see text footnote 1].

It is recognized that protein combinations, such as fusion proteins, as well as the expression of these proteins by different expression vectors are important strategies in the development of rBCG vaccines with an enhanced efficacy compared with BCG. Nevertheless, the vaccine approach of super expressing Mtb proteins in BCG, such as rBCG:30, which expresses only Ag85B, to induce central memory and more desirable protection than BCG, is useful (49). This plasmid-based vaccine passed a phase I clinical trial and is currently on hold awaiting the development of auxotrophic BCG strains to avoid the use of antibiotic resistance genes (see text footnote 1).

An intriguing point is that an rBCG vaccine currently in a phase II human clinical trial does not contain Mtb antigens or antigens lost by BCG during the attenuation process. The rBCGΔureC:hly vaccine improved BCG antigen presentation by dendritic cells and improved processing with the ultimate goal of activating CD8+ T cells. Therefore, this approach might have overcome some of the evolutionary mycobacteria immunological escape mechanisms and will allow protective long-lasting functional memory. In time, whether this vaccine induced better protection against TB will be determined (77).

Furthermore, the choice of parental BCG strain appears not to interfere with the recombinant vaccine outcome, because some vaccines using the same parental BCG strains had different outcomes depending on the selected antigen or fusion protein used. Likewise, the immune response profile of those vaccine candidates that showed better protection than BCG was based upon CD4 and CD8 T cells with polyfunctional activities. From all studies reviewed here, only six successfully evaluated immunological memory in animal models.

The animal models available to study TB vaccines (mice, guinea pig, or non-human primates) cannot predict the outcome among vaccinated humans. It is well-known that mice and guinea pigs are infected by BCG vaccination and the duration of the vaccination and the time until challenge are crucial to address the persistence of memory T cells. This premise could be used to justify the low number of studies that have addressed this issue over the past 5 years.

The real impact of these new vaccines using rBCG or other strategies that are currently in clinical trials will only be determined 5–10 years from now. Therefore, studies addressing new strategies to improve BCG need to be continued.

## Materials and Methods

### Study selection and data collection process

The search for this review was conducted in April 2013, and was based on articles published in the previous 5 years (2008–2013). Articles were searched from the PubMed Database using the following key words: tuberculosis protection and rBCG vaccine with the intention to address publications showing studies on rBCG vaccine for tuberculosis. Then further manuscripts were selected using the key words: tuberculosis protection; rBCG vaccine and memory. Manuscripts lacking information of the BCG wild type strain and those that used the boost strategy without evaluating rBCG responses alone were not included in this review.

## Conflict of Interest Statement

The authors declare that the research was conducted in the absence of any commercial or financial relationships that could be construed as a potential conflict of interest.

## Footnotes

^1^www.clinicaltrials.com
